# Efficacy of cisplatin-gemcitabine-durvalumab in patients with advanced biliary tract cancer experiencing early vs late disease relapse after surgery: a large real-life worldwide population

**DOI:** 10.1093/oncolo/oyae256

**Published:** 2024-10-19

**Authors:** Federica Lo Prinzi, Francesca Salani, Margherita Rimini, Mario Domenico Rizzato, Lorenzo Antonuzzo, Silvia Camera, Tomoyuki Satake, Hanne Vandeputte, Caterina Vivaldi, Tiziana Pressiani, Jessica Lucchetti, Jin Won Kim, Oluseyi Abidoye, Ilario Giovanni Rapposelli, Stefano Tamberi, Fabian Finkelmeier, Guido Giordano, Chiara Pircher, Hong Jae Chon, Chiara Braconi, Alessandro Pastorino, Florian Castet, Emiliano Tamburini, Changhoon Yoo, Alessandro Parisi, Anna Diana, Mario Scartozzi, Gerald W Prager, Antonio Avallone, Marta Schirripa, Il Hwan Kim, Lukas Perkhofer, Ester Oneda, Monica Verrico, Jorge Adeva, Stephen L Chan, Gian Paolo Spinelli, Nicola Personeni, Ingrid Garajova, Maria Grazia Rodriquenz, Silvana Leo, Cecilia Melo Alvim, Ricardo Roque, Lorenzo Fornaro, Antonio De Rosa, Daniele Lavacchi, Federico Rossari, Masafumi Ikeda, Jeroen Dekervel, Monica Niger, Rita Balsano, Giuseppe Tonini, Minsu Kang, Tanios Bekaii-Saab, Massimo Giuseppe Viola, Lucrezia Silvestro, Luca Esposito, Alessandra Boccaccino, Vera Himmelsbach, Matteo Landriscina, Selma Ahcene Djaballah, Valentina Zanuso, Gianluca Masi, Sara Lonardi, Lorenza Rimassa, Andrea Casadei-Gardini

**Affiliations:** Operative Research Unit of Medical Oncology, Fondazione Policlinico Universitario Campus Bio-Medico, Via Alvaro del Portillo, 200 - 00128 Roma, Italy; Unit of Medical Oncology 2, Azienda Ospedaliero-Universitaria Pisana, 56126 Pisa, Italy; Department of Translational Research and New Technologies in Medicine and Surgery, University of Pisa, 56126 Pisa, Italy; Department of Oncology, Vita-Salute San Raffaele University, IRCCS San Raffaele Scientific Institute Hospital, 20132 Milan, Italy; Department of Oncology, Veneto Institute of Oncology IOV - IRCCS, 35128 Padua, Italy; Clinical Oncology Unit, Department of Experimental and Clinical Medicine, Careggi University Hospital, University of Florence, 50134 Florence, Italy; Thoracic Surgery Unit, Department of Experimental and Clinical Medicine, Careggi University Hospital, University of Florence, 50134 Florence, Italy; Department of Oncology, Vita-Salute San Raffaele University, IRCCS San Raffaele Scientific Institute Hospital, 20132 Milan, Italy; Department of Hepatobiliary and Pancreatic Oncology, National Cancer Center Hospital East, Kashiwa 277-8577, Japan; Digestive Oncology, University Hospitals Leuven, 3000 Leuven, Belgium; Unit of Medical Oncology 2, Azienda Ospedaliero-Universitaria Pisana, 56126 Pisa, Italy; Department of Translational Research and New Technologies in Medicine and Surgery, University of Pisa, 56126 Pisa, Italy; Medical Oncology and Hematology Unit, Humanitas Cancer Center, IRCCS Humanitas Research Hospital, Rozzano, 20089 Milan, Italy; Operative Research Unit of Medical Oncology, Fondazione Policlinico Universitario Campus Bio-Medico, Via Alvaro del Portillo, 200 - 00128 Roma, Italy; Division of Hematology/Medical Oncology, Department of Internal Medicine, Seoul National University Bundang Hospital, Seoul National University College of Medicine, Gumi-ro 173 Beon-gil, Bundang-gu, Seongnam-si, Gyeonggi-do 13620, Republic of Korea; Department of Internal Medicine, Mayo Clinic, Phoenix, AZ 5777, United States; Department of Medical Oncology, IRCCS Istituto Romagnolo per lo Studio dei Tumori (IRST) “Dino Amadori”, 47014 Meldola, Italy; Medical Oncology, Santa Maria delle Croci hospital, Ravenna AUSL, 48121 Romagna, Italy; Medical Clinic 1, Department of Gastroenterology, University Hospital Frankfurt, 60596 Frankfurt am Main, Germany; Unit of Medical Oncology and Biomolecular Therapy, Policlinico Riuniti, 71122 Foggia, Italy; Department of Medical and Surgical Sciences, University of Foggia, 71122 Foggia, Italy; Department of Medical Oncology, Fondazione IRCCS Istituto Nazionale dei Tumori, 20133 Milan, Italy; Division of Medical Oncology, Department of Internal Medicine, CHA Bundang Medical Center, CHA University School of Medicine, Seongnam 59, South Korea; University of Glasgow (School of Cancer Sciences), Beatson West of Scotland Cancer Centre, CRUK Scotland Centre, Glasgow G61 1BD, United Kingdom; IRCCS Ospedale Policlinico San Martino, Medical Oncology Unit 1, 16132 Genova, Italy; Gastrointestinal and Endocrine Tumor Unit, Vall d’Hebron Institute of Oncology (VHIO), Hospital Universitari Vall d’Hebron, Vall d’Hebron Barcelona Hospital Campus, 08035 Barcelona, Spain; Department of Oncology and Palliative Care, Cardinale G Panico, Tricase City Hospital, 73039 Tricase, Italy; ASAN Medical Center, University of Ulsan College of Medicine, Seoul 138-736, Republic of Korea; Clinica Oncologica e Centro Regionale di Genetica Oncologica, Università Politecnica delle Marche, Azienda Ospedaliero-Universitaria delle Marche, Via Conca 71, 60126 Ancona, Italy; Oncology Unit, Ospedale del Mare, 80147 Napoli, Italy; Medical Oncology, University and University Hospital, 09124 Cagliari, Italy; Department of Medicine I, Clinical Division of Oncology, Medical University of Vienna, 1090 Vienna, Austria; Experimental Clinical Abdominal Oncology Unit, Istituto Nazionale Tumori – IRCCS - Fondazione G. Pascale, 80131 Naples, Italy; Medical Oncology Unit, Department of Oncology and Hematology, Belcolle Hospital, 01100 Viterbo, Italy; Division of Oncology, Department of Internal Medicine, Haeundae Paik Hospital, Inje University College of Medicine, Busan 875, Republic of Korea; Internal Medicine 1, University Hospital Ulm, 89081 Ulm, Germany; Institute of Molecular Oncology and Stem Cell Biology, Ulm University Hospital, 89081 Ulm, Germany; Dipartimento di Oncologia medica, Fondazione Poliambulanza, 25124 Brescia, Italy; UOC Oncologia A, Department of Hematology, Oncology and Dermatology, Policlinico Umberto I University Hospital, Sapienza University o f Rome, Viale Regina Elena, 324, 00161 Rome, Italy; 12 de Octubre University Hospital, Spanish Society of Medical Oncology (SEOM), 28041 Madrid, Spain; State Key Laboratory of Translational Oncology, Department of Clinical Oncology, Prince of Wales Hospital, The Chinese University of Hong Kong, Hong Kong 30, China; UOC Oncologia Territoriale, Polo Pontino, La Sapienza Università Di Roma, 04100 Latina, Italy; Medical Oncology Unit, P.O. Manerbio - ASST Garda, 25025 Manerbio, Brescia,Italy; Medical Oncology Unit, University Hospital of Parma, 43126 Parma, Italy; Oncology Unit, Fondazione IRCCS “Casa Sollievo della Sofferenza”, 71013 San Giovanni Rotondo, Italy; Division of Oncology, Vito Fazzi Hospital, 73100 Lecce, Italy; Medical Oncology Department, Hospital de Santa Maria, Centro Hospitalar Universitário Lisboa Norte, 1649-035 Lisbon, Portugal; Portuguese Institute of Oncology of Coimbra, 3000-075 Coimbra, Portugal; Unit of Medical Oncology 2, Azienda Ospedaliero-Universitaria Pisana, 56126 Pisa, Italy; Department of Oncology, Veneto Institute of Oncology IOV - IRCCS, 35128 Padua, Italy; Department of Surgery, Oncology and Gastroenterology, University of Padua, 35121 Padua, Italy; Clinical Oncology Unit, Department of Experimental and Clinical Medicine, Careggi University Hospital, University of Florence, 50134 Florence, Italy; Department of Oncology, Vita-Salute San Raffaele University, IRCCS San Raffaele Scientific Institute Hospital, 20132 Milan, Italy; Department of Hepatobiliary and Pancreatic Oncology, National Cancer Center Hospital East, Kashiwa 277-8577, Japan; Digestive Oncology, University Hospitals Leuven, 3000 Leuven, Belgium; Department of Medical Oncology, Fondazione IRCCS Istituto Nazionale dei Tumori, 20133 Milan, Italy; Medical Oncology and Hematology Unit, Humanitas Cancer Center, IRCCS Humanitas Research Hospital, Rozzano, 20089 Milan, Italy; Department of Biomedical Sciences, Humanitas University, 20072 Pieve Emanuele (Milan), Italy; Department of Medicine and Surgery, Università Campus Bio-Medico di Roma, Via Alvaro del Portillo, 21 - 00128 Roma, Italy; Division of Hematology/Medical Oncology, Department of Internal Medicine, Seoul National University Bundang Hospital, Seoul National University College of Medicine, Gumi-ro 173 Beon-gil, Bundang-gu, Seongnam-si, Gyeonggi-do 13620, Republic of Korea; Department of Internal Medicine, Mayo Clinic, Phoenix, AZ 5777, United States; Department of Oncology and Palliative Care, Cardinale G Panico, Tricase City Hospital, 73039 Tricase, Italy; Experimental Clinical Abdominal Oncology Unit, Istituto Nazionale Tumori – IRCCS - Fondazione G. Pascale, 80131 Naples, Italy; Department of Medical Oncology, IRCCS Istituto Romagnolo per lo Studio dei Tumori (IRST) “Dino Amadori”, 47014 Meldola, Italy; Medical Oncology, Santa Maria delle Croci hospital, Ravenna AUSL, 48121 Romagna, Italy; Medical Clinic 1, Department of Gastroenterology, University Hospital Frankfurt, 60596 Frankfurt am Main, Germany; Unit of Medical Oncology and Biomolecular Therapy, Policlinico Riuniti, 71122 Foggia, Italy; Department of Medical and Surgical Sciences, University of Foggia, 71122 Foggia, Italy; Department of Oncology, Veneto Institute of Oncology IOV - IRCCS, 35128 Padua, Italy; Medical Oncology and Hematology Unit, Humanitas Cancer Center, IRCCS Humanitas Research Hospital, Rozzano, 20089 Milan, Italy; Department of Biomedical Sciences, Humanitas University, 20072 Pieve Emanuele (Milan), Italy; Unit of Medical Oncology 2, Azienda Ospedaliero-Universitaria Pisana, 56126 Pisa, Italy; Department of Translational Research and New Technologies in Medicine and Surgery, University of Pisa, 56126 Pisa, Italy; Department of Oncology, Veneto Institute of Oncology IOV - IRCCS, 35128 Padua, Italy; Medical Oncology and Hematology Unit, Humanitas Cancer Center, IRCCS Humanitas Research Hospital, Rozzano, 20089 Milan, Italy; Department of Biomedical Sciences, Humanitas University, 20072 Pieve Emanuele (Milan), Italy; Department of Oncology, Vita-Salute San Raffaele University, IRCCS San Raffaele Scientific Institute Hospital, 20132 Milan, Italy

**Keywords:** cholangiocarcinoma, durvalumab, immunotherapy, real-world evidence, biliary tract cancer, advanced disease, surgery

## Abstract

**Background:**

In the TOPAZ-1, patients with biliary tract cancers (BTC) and recurrence within 6 months after surgery were excluded, even if this event is frequently observed in clinical practice. Our study aimed to assess if the efficacy of cisplatin-gemcitabine-durvalumab (CGD) in this population is comparable to that reported in the phase 3 trial.

**Methods:**

The study cohort included patients with BTC who underwent surgery on the primary tumor, experienced disease recurrence occurring ≤6 months or >6 months after surgery or after the end of adjuvant therapy and started CGD. The primary objectives were overall survival (OS) and progression free survival (PFS).

**Results:**

A total of 178 patients were enrolled. No significant differences were observed between early and late relapse groups in OS (23.4 months vs not reached; HR 1.26; 95% CI, 0.67-2.37; *P* = .45) and PFS [7.0 months vs 9.8 months; HR 1.3(95% CI, 0.9-2.1) *P* = .13]. Overall response rate and disease control rate (*P* = .33 and *P* = .62) were comparable between the 2 groups, as the overall safety profile. In addition, we compared survival outcomes between the selected population and a historical cohort of patients with BTC treated with cisplatin-gemcitabine (CG) and found that despite the absence of statistical significance, CGD showed an outcome trend compared with CG regardless of the time of recurrence after surgery or adjuvant chemotherapy [(CG ≤ 6 vs CGD ≤ 6 months: HR 0.59, 95%CI, 0.35-1.01, *P* = .05; HR 0.70; 95%CI, 0.46-1.06, *P* = .09, OS and PFS, respectively) and (CG > 6 vs. CGD > 6 months: HR 0.50; 95%CI, 0.29-0.88, *P* = 0.0165; HR 0.54; 95%CI, 0.35-0.84, *P* = .0068, OS and PFS, respectively)].

**Conclusion:**

Our analysis suggests that CGD retains its efficacy independently of the timing of relapse after surgery or completion of adjuvant treatment in patients with advanced BTC.

## Teaser

This article reports results of first-line cisplatin-gemcitabine-durvalumab in patients with biliary tract cancer with disease recurrence within 6 months after surgery or completion of adjuvant therapy, showing that this group of patients can benefit from chemoimmunotherapy in terms of survival outcomes.

**Implications for practice**

Our analysis reports for the first time the actual results of first-line CGD in patients with biliary tract cancers with disease recurrence within 6 months after surgery or completion of adjuvant therapy, showing that this group of patients, similar to the group with recurrence and initiation of systemic therapy after 6 months, can benefit from chemoimmunotherapy in terms of survival outcomes, without a significant difference in toxicity.

## Introduction

Gallbladder cancer (GBC), intrahepatic and extrahepatic (distal, peri-hilar) cholangiocarcinoma (CCA) are collectively known as biliary tract cancers (BTCs).^1^ BTCs have been considered rare cancers: nevertheless, the incidence is increasing, mainly if referring to intrahepatic cholangiocarcinoma (iCCA).^2^ BTCs are known to have a poor prognosis, with an estimated 5-year overall survival (OS) rate of <20% when all stages are analyzed together.^2^ This is attributable to the high rate of diagnosis at advanced stages when the disease is mainly managed by palliative therapeutic approaches. In addition, for those patients diagnosed at early stages, the relapse rate remains at approximately 70%.^2^

The only treatment with curative intent is surgery, which is the current gold standard for patients with resectable disease.^3^ However, relapse rates remain high, particularly for patients with node-positive disease or microscopically involved margins (R1).^4^ To identify patients undergoing radical-intent surgery with a higher risk of very early recurrence (VER, defined as recurrence within 6 months after surgery), an online VER calculator was formulated, to help physicians select patients at higher risk of VER after surgery.^5^ Following curative resection, current evidence supports the role of adjuvant chemotherapy with fluoropyrimidines for 6 months based on the phase III BILCAP and JCOG1202, ASCOT trials.^6-8^

In the setting of advanced disease, cisplatin-gemcitabine-durvalumab (CGD) or cisplatine-gemcitabine-pembrolizumab are the 2 current options for the first-line standard of care based on the survival benefit shown in the phase III randomized placebo-controlled TOPAZ-1 and KEYNOTE-966 studies.

The phase III TOPAZ-1 trial, a randomized double-blind placebo-controlled study, evaluated the addition of the anti-programmed cell death ligand 1 (PD-L1) antibody durvalumab to chemotherapy with cisplatin and gemcitabine in the first line setting and demonstrated a survival advantage for the combination of durvalumab and chemotherapy over chemotherapy alone.^9,10^ Similarly, the phase III KEYNOTE-966 study showed a significant improvement in overall survival (OS) for patients receiving pembrolizumab in combination with cisplatin and gemcitabine compared to those receiving cisplatin and gemcitabine alone.^11,12^

Recently, several new therapeutic possibilities have emerged thanks to novel molecular insights highlighting a number of potential targets, some of which have already been investigated in phase III trials.^13-18^

In the TOPAZ-1 study, patients with previously untreated, unresectable, or metastatic disease at diagnosis, as well as those who developed disease recurrence more than 6 months after surgery or more than 6 months after completing adjuvant therapy were enrolled. Patients who developed recurrence less than 6 months after surgery or or less than 6 months after completion of adjuvant therapy were excluded.

However, in clinical practice, patients frequently relapse during the first 6 months after surgery or during the first 6 months after the completion of adjuvant therapy. Therefore, it is important to understand if this group of patients can obtain the same benefit from CGD. Currently, the TOURMALINE phase IIIb trial is enrolling participants to evaluate the efficacy and safety of combining durvalumab with different first-line chemotherapy regimens and also allows the inclusion of patients with early relapsed BTC.^19^ The present study aims to assess in a large real-world, international setting, if there are significant differences in terms of efficacy and safety between patients who underwent surgery on the primary tumor, experienced disease recurrence occurring ≤6 months or >6 months after surgery or completion of adjuvant therapy and started first-line therapy with CGD.

## Material and methods

### Study population

The study population included radically resected BTC (including iCCA, eCCA, and GBC) with recurrence in form of unresectable, locally advanced, or metastatic BTC, and treated with CGD in the first-line setting. Data were collected retrospectively, but the participants were enrolled consecutively from 41 sites across 12 countries (Italy, Germany, Austria, Spain, Portugal, Belgium, United Kingdom, United States, Republic of Korea, China, Hong Kong Special Administrative Region of China, and Japan). Patients were treated with CGD administered intravenously on a 21-day cycle for up to 8 cycles. Durvalumab (1500 mg) was administered on day 1 of each cycle, in combination with gemcitabine (1000 mg/m^2^) and cisplatin (25 mg/m^2^), which were administered on days 1 and 8 of each cycle. After completion of gemcitabine and cisplatin, durvalumab monotherapy (1500 mg) was administered every 4 weeks until disease progression or unacceptable toxicity.

The population tested was stratified into 2 groups: the early relapse group (recurrence less than 6 months after surgery or less than 6 months after completion of adjuvant therapy) and late relapse group (disease relapse >6 months after surgery or >6 months after the completion of adjuvant therapy) both starting CGD in that period. Patients who did not undergo surgery on the primary tumor due to locally advanced or metastatic disease at diagnosis were excluded from the analysis.

Besides, to confirm the efficacy of adding durvalumab to chemotherapy in patients with BTC, we compared the population selected and treated with CGD with a historical cohort of patients treated with CG. Data were collected retrospectively, but the participants were enrolled consecutively from seventeen centers in Italy from March 2006 to December 2022. Patients who received treatment before the publication of the TOPAZ-1 results received the previous standard combination of cisplatin 25 mg/m^2^ plus gemcitabine 1000 mg/m^2^ on days 1 and 8 of each 21-day cycle for up to 8 cycles, according to the ABC-02 trial. This cohort comprised 111 individuals with advanced BTC whose 59 patients had an early relapse and 52 patients had a late relapse.

The present study was approved by the local Ethics Committee at each center, complied with the provisions of the Good Clinical Practice guidelines and the Declaration of Helsinki and local laws, and fulfilled the Regulation (EU) 2016/679 of the European Parliament and of the Council of 27 April 2016 on the protection of natural persons with regard to the processing of personal data.

### Statistical analysis

The analysis aimed to determine whether there were differences in survival outcomes (OS and PFS) between the groups with early and late recurrence. OS was defined as the time from the beginning of first-line therapy to death from any cause. PFS was defined as the time from the beginning of the first line therapy to disease progression or death. OS was estimated by the Kaplan-Meier method and curves were compared by the log-rank test. Unadjusted and adjusted hazard ratios (HRs) by baseline characteristics were calculated using the Cox proportional hazards model. A *P* value < .05 was considered statistically significant. The median time to relapse was calculated as that time from surgery to recurrence of the disease. Patients were followed every 2-3 months with a multiphasic scanning technique, based on the clinical practice of the center. Treatment response data was extracted from the local radiological evaluation and categorized as complete response (CR), partial response (PR), stable disease (SD), or progressive disease (PD) according to Response Evaluation Criteria in Solid Tumors (RECIST) 1.1. ORR was defined as the proportion of patients who achieved CR or PR. Disease control rate (DCR) was defined as the proportion of patients who achieved CR, PR, or SD.

Adverse events (AEs) were graded according to the National Cancer Institute Common Terminology Criteria for Adverse Events, version 5.0.

MedCalc package (MedCalc® version 16.8.4) was used for statistical analysis.

## Results

### Patients

The initial population consisted of 666 patients included in our previous studies. Of them, 178 patients underwent surgery and were included in our analysis; 488 patients were excluded from the analysis, they were all metastatic from diagnosis and had not undergone surgery on the primary tumor while the included patients were 91 patients (51%) had a late relapse and 87 patients (48%) had an early relapse; 46 patients (52.8%) who experienced early relapse received adjuvant therapy, with 41 (89.1%) patients were treated with Capecitabine according to the BILCAP trial; 63 patients (69.2%) who experienced late relapse received adjuvant therapy, with 55(87.3%) patients were treated with Capecitabine. The 69 (38.7%) remaining patients in the early and late relapse groups did not receive adjuvant therapy, mainly due to recurrence within one month after surgery and having an early stage. Patient characteristics are listed in Table 1

**Table 1.** Patient characteristics.

Characteristic	Relapse and started systemic therapy ≤ 6 months *N* (%) *N* = 87	Relapse and started systemic therapy > 6 months *N* (%) *N* = 91	*P*
*Gender*			
Male	56(64.3)	48(52.7)	.13
Female	31 (35.6)	43(49.4)	
Age	68 (range 34-85)	69 (range 47-91)	
>/=70	43 (49.4)	44(48.35)	1.00
<70	44 (50.5)	46(50.5)	
Not reported	2(2.2)	1(1.09)	
*Primary tumor site*			
Intrahepatic	34(39)	28(30.7)	.009
Extrahepatic	25(60.9)	46(69.2)	
Gallbladder	28(32.1)	17(18.68)	
*Stage AJCC*			
I-II	19(21.8)	48(52.7)	.000006
III-IV	51(58.6)	26(28.5)	
Not reported	17(19.5)	17(18.6)	
*Drainage or stent*			<.000001
Yes	15(17.2)	76(81.5)	
No	72 (82.7)	15(16.4)	
*ECOG PS*			
0	53 (60.9)	61 (67)	.4
>0	34 (39)	30 (32.9)	
CA 19-9 median (range) UI/mL	111(1-1700)	105 (1-20 976)	
Within normal levels	37 (42.5)	31(34)	.3
>Normal levels	45 (51.7)	53(60.4)	
Not reported	5 (5.74)	7(7.6)	
CEA median (range) ng/mL	3.1 (0.5-681)	3.14 (0.3-3594)	
Within normal levels	44 (50.5)	54(59.3)	.13
>Normal levels	34 (39)	25(27.4)	
Not reported	9(10.3)	12(13.1)	
NLR			
<3	51(58.6)	44(48.3)	.2
≥3	28(32.1)	37 (40.6)	
Not reported	8(9.1)	10(10.9)	
*Albumine g/dL*			
Within normal levels	42(48.2)	52(57.1)	.4
<Normal levels	11(12.6)	9(9.8)	
Not reported	34(39)	30(32.9)	
*Adjuvant therapy*			
Yes	46(52.8)	63 (69.2)	.03
No	41(47.1)	28 (30.7)	
Scheme of adjuvant therapy			
Capecitabine according to BILCAP trial	41(89.1)	55 (87.3)	1.0
Others	5(19.8)	8(12.6)	
*Kind of relapse*			
Local	17 (19.5)	20 (21.9)	.7
Metastatic	70 (80.4)	71 (78.0)	

Abbreviations: ECOG PS, Eastern Cooperative Oncology Group performance status; NLR, neutrophil to lymphocyte ratio; Stage AJCC, the American Joint Committee on Cancer.

There were almost no significant differences in the baseline characteristics between both subgroups. The late relapse group had a higher number of biliary stent placements (81.5% vs 17.2%; *P* = <.000001), while the early relapse group had a higher number of cases with a stage III-IV (T4N0M0) at previous surgery (51% vs 26%; *P* = .000006).

### Efficacy and safety of CGD

At the data cutoff (July 31, 2023), the median duration of follow-up was 9.6 months (95% CI:7.6-10.8), 86 patients (48.3%) discontinued treatment due to disease progression, and 39 patients (22.0%) died. In the entire population, the median OS was 23.4 months (95%CI 14.6-23.4) and the median PFS was 8.9 months (95%CI 7.9-9.9).

The median time to relapse was 2.8 months (range, 0.05-5.95 months) for the early relapse group and 15.5 months (range, 6.03-99.89 months) for the late relapse group.

**Figure 1.**
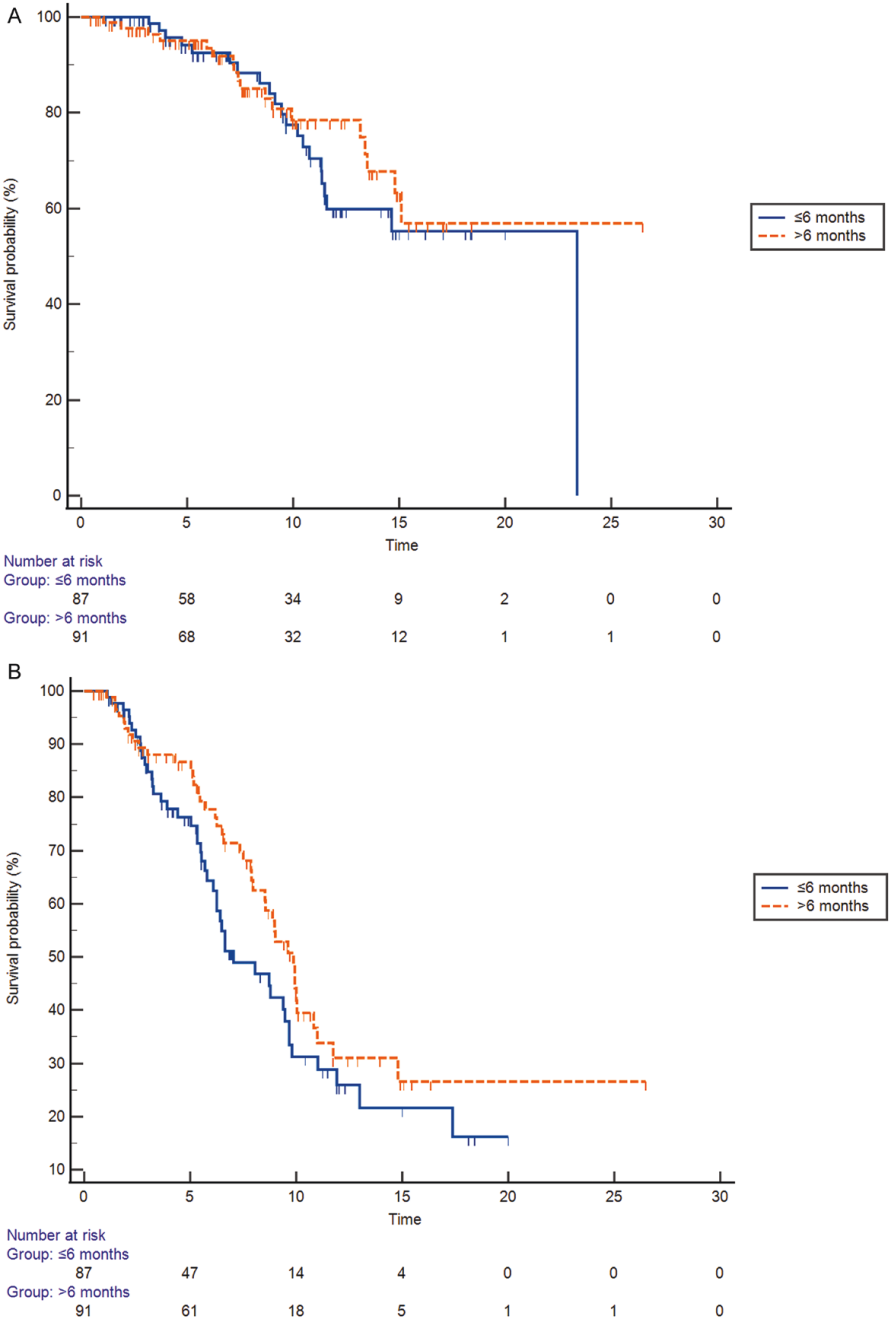
a: Kaplan-Meier curves of OS in patients with disease relapse ≤6 months after surgery/completion of adjuvant therapy and those with disease relapse >6 months after surgery/completion of adjuvant therapy. b: Kaplan-Meier curves of PFS in patients with disease relapse ≤6 months after surgery/completion of adjuvant therapy and those with disease relapse >6months after surgery/completion of adjuvant therapy.

At univariate analysis, no significant differences were found between early vs late relapse groups in terms of OS [(23.4 months vs not reached; HR 1.26; 95% CI, 0.67-2.37; *P* = .45)] (Figure 1A) and PFS [(7.0 months (95% CI, 6.0-9.6) vs 9.8 months (95% CI, 7.9-10.8); HR 1.38 (95% CI, 0.90-2.13) *P* = .13] (Figure 1B), respectively. Among the studied variables, ECOG PS > 0 (HR 2.8; 95% CI, 1.38-5.66; *P* = .004), CA 19-9 > baseline normal levels (HR 2.19; 95% CI, 1.11-4.31; *P* = .02), CEA > baseline normal levels (HR 2.07; 95% CI, 1.03-4.19; *P* = .04), NLR > 3 (HR 2.02; 95% CI, 1.05-3.89; *P* = .03), and albumin < baseline normal levels (HR 3.73; 95% CI 1.32-10.56; *P* = 0.012) were associated with poorer OS at univariate analysis (Table 2). ECOG PS > 0 (HR 2.04; 95% CI, 1.25-3.31; *P* = .003) and CA 19-9 >baseline normal levels (HR 1.99; 95%CI, 1.27-3.12; *P* = .002) were prognostic for shorter PFS at univariate analysis (Table 2).

**Table 2.** Univariate and multivariate analysis of OS and PFS.

	OS						PFS					
	Univariate			Multivariate			Univariate			Multivariate		
Parameters	HR	95% CI	*P*	HR	95% CI	*P*	HR	95% CI	*P*	HR	95% CI	*P*
*Age*												
>70	1						1					
≤70	1.19	0.62-2.28	.58				1.06	0.69-1.63	.77			
*Gender*												
Male	1						1					
Female	1.06	0.56-2.03	.83				1.16	0.74-1.80	.50			
*Primary tumor Site*												
iCCA	1			1			1			1		
Gallbladder	0.91	0.80-4.55	.03	2.16	0.72-6.4	.16	1.27	0.70-2.28	.46	0.90	0.39-2.07	.80
eCCA	0.72	0.35-1.4	.03	0.45	0.11-1.8	.27	0.91	0.56-1.48	.46	0.59	0.25-1.36	.21
*Biliary drainage*												
Yes	1						1					
No	1.12	0.52-2.40	.76				1.38	0.81-2.35	.22			
*ECOG PS*												
>0	2.80			2.57			2.04			2.1		
=0	1	1.38-5.66	.004	1	1.00-6.67	.04	1	1.25-3.31	.003	1	1.05-4.30	.034
*CA 19-9*												
>Normal levels	2.19	1.11-4.31	.02	0.52	0.17-1.53	.23	1.99	1.27-3.12	.002	3.06	1.39-6.71	.005
*Normal levels*	1			1			1			1		
CEA												
>Normal levels	2.07	1.03-4.19	.04	1.28	0.50-3.31	.59	1.36	0.84-2.19	.20	0.97	0.50-1.88	.94
Norma levels	1			1			1		1			
*Relapse and start CGD*												
<6 months	1.26	0.67-2.37	.45	0.53	0.19-1.48	.23	1.38 1	0.90-2.13	.13	1.11	0.53-2.32	.76
>6 months	1			1						1		
*NLR*												
>3	2.02	1.05-3.89	.03	1.50	0.59-3.82	.39	1.08	0.69 to 1.70	.71	1.07	0.55-2.09	.83
≤3	1			1			1			1		
*Albumine*												
>Normal levels	3.73	1.32-10.56	.012	2.91	0.93-9.05	.06	1.86	0.88-3.93	.09	2.29	0.96-5.45	.05
Normal levels	1			1			1			1		
Stage AJCC												
I-II	1						1					
III-IV	2.07	0.99-4.31	.05				1.10	0.68-1.79	.68			
*Adjuvant therapy in early relapse group*												
Yes	0.63	0.25-1.55	.31				0.89	0.49-1.62	.77			
No	1						1					
*Adjuvant therapy in late group*												
Yes	0.44	0.16-1.19	.10				1.07	0.63-1.83	.77			
No							1					

Abbreviations: CGD, Cisplatin-Gemcitabine-Durvalumab; OS, overall survival; PFS, progression free survival.

Multivariate analysis confirmed the absence of significant differences between early and late relapse groups in terms of OS (HR 0.53; 95% CI, 0.19-1.48; *P* = .23) and PFS (HR 1.11; 95% CI, 0.53-2.32; *P* = .76) (Table 2). Furthermore, multivariate analysis for OS confirmed that patients with ECOG PS > 0 had significantly shorter OS (HR 2.57; 95% CI, 1.00-6.67; *P* = .04). The multivariate analysis of PFS confirmed the association of ECOG PS > 0 (HR 2.1; 95% CI, 1.05-4.30, *P* = .034) and CA 19-9 > baseline normal levels (HR 3.06; 95%CI, 1.39-6.71, *P* = .005) with shorter PFS (Table 2).

No differences were reported in terms of ORR, DCR (*P* = .33 and *P* = .62, respectively) nor safety between the 2 groups (Tables S1 and 2). Immunotherapy-related adverse events such as thyroid function changes, skin rash, renal function changes, and cholangitis were all found to be in equal frequency between the two groups as were the most common chemotherapy-related side effects.

### Efficacy of CGD compared to CG

**Figure 2.**
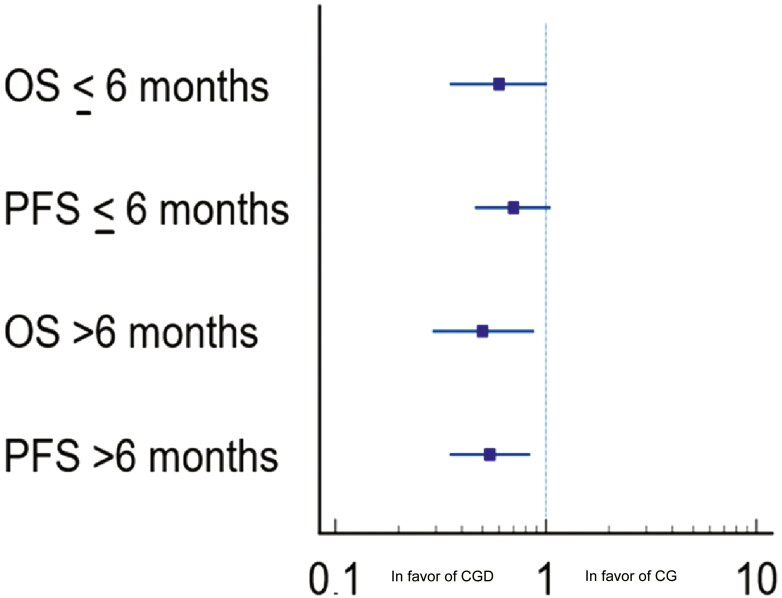
Forest plot of OS and PFS in patients with disease relapse and treatment initiation [cisplatin-gemcitabine (CG) and cisplatin-gemcitabine-durvalumab (CGD)] ≤6 months after surgery/completion of adjuvant therapy, and OS and PFS in patients with disease relapse and treatment initiation [cisplatin-gemcitabine and cisplatin-gemcitabine-durvalumab) >6 months after surgery/completion of adjuvant therapy.

To evaluate the efficacy of adding durvalumab to chemotherapy, we compared the CGD cohort with a historical cohort of patients treated with CG. This cohort comprised 111 individuals with advanced BTC: 59 patients had an early relapse and 52 patients had late relapse. Figure 2 presents the forest plots of OS and PFS between CG and CGD in the early relapse subset (HR 0.59; 95%CI, 0.35-1.01, *P* = .05; HR 0.70; 95%CI, 0.46-1.06, *P* = .09, respectively) and in the late relapse subset (HR 0.50; 95%CI, 0.29-0.88, *P* = .0165; HR 0.54; 95%CI, 0.35-0.84, *P* = .007, respectively).

No differences in terms of ORR and DCR were reported between patients treated with CG and CGD in the early relapse subset [ORR 30.5% vs 29.8%; DCR 62.7% vs 68.9% (*P* = 1.0 and *P* = 0.47, respectively)] neither in the late relapse subset [ORR 38.4%vs 30.7%; DCR 63.4% vs 72.5% (*P* = .36 and *P* = .26, respectively)] (Table S3a-3b).

## Discussion

In the present analysis, we first reported that the survival benefit and overall safety of CGD are not affected by the timing of relapse after surgery or completion of adjuvant treatment. Therefore, our analysis suggests that even patients with early relapse (ie, within 6 months) who were excluded from the TOPAZ-1 study might be safely treated and could benefit from the addition of durvalumab to CG.

As shown in our analysis, patients with early relapse had a higher proportion of advanced-stage surgery (stage III). In contrast, the group of patients with late relapse had a higher proportion of biliary stent placements: this is probably because the majority of patients in this group had a diagnosis of eCCA, which is frequently associated with biliary obstruction and jaundice.^20^ These observations reassure us about the overall efficacy and safety profile of CGD even in a worse prognosis subgroup. Indeed, the adverse events were comparable in the 2 subgroups.

Of note, including patients with early relapse, who were considered to have a worse prognosis, we observed a longer median OS (23.3 months) compared to that reported in TOPAZ-1 study (12.8 months). We could speculate, on one hand, that early relapse after initial treatment could be associated with higher cellular turnover and consequently greater inflammatory infiltrate: this could establish a tumor microenvironment that is highly reactive to chemo-immunotherapy compared to those observed in patients with more indolent disease.^21^ On the other hand, the long OS of our cohort could be influenced by primary surgery. Previous studies have suggested that resection of the primary tumor may have an impact on future outcomes. A retrospective analysis revealed that patients who underwent primary resection had a longer median survival compared to patients with unresectable disease at diagnosis and candidate for palliative treatment (27.6 months vs 12.9 months, *P* < .001).^22^ Also, in a cohort of 864 patients with BTC treated with palliative chemotherapy, the lack of previous surgery was considered an independent negative prognostic factor.^23^ The observation that patients experiencing early relapse could nonetheless benefit from CGD is, in our opinion, clinically relevant. Indeed, it should be considered that the percentage of patients with BTC eligible for surgery is approximately 25%, and the recurrence rate remains high after surgery.^24^ There is no consensus on the exact timing of early recurrence among patients with BTC. Many patients experience early recurrence in the first months following resection. In a study of 880 patients, approximately 22.3% experienced recurrence within 6 months of resection.^5^ Therefore, a high percentage of patients are at risk of being excluded from CGD if TOPAZ-1 selection criteria are strictly applied. Moreover, our analysis suggests that patients with early relapse could be considered for future studies of first-line treatment. Currently, the TOURMALINE phase IIIb study is underway to evaluate the safety and efficacy of durvalumab in combination with different gemcitabine-based chemotherapy regimens as first-line therapy for patients with BTC. Unlike TOPAZ-1, patients with early relapse were not excluded. The results of this study will be important to further clarify the potential benefit of systemic therapy for patients who experience an early relapse in a prospective setting.^19^

To further confirm the benefit of using the durvalumab in patients experiencing early relapse, we also performed a comparison analysis between patients receiving CGD and a historical cohort of patients treated with CG. This analysis confirmed an OS and PFS benefit in favor of the durvalumab-containing regimen in the late relapse group (further confirming the results of TOPAZ-1 in a real-world setting), with a convincing trend also in the early relapse subset. The lack of statistical significance in this latter group may be due to the limited sample size. Taken together, our results suggest that the timing of relapse after surgery or adjuvant therapy seems not to impact the efficacy of durvalumab when added to CG.

In our study, we also confirmed that some patient or disease characteristics are associated with worse outcomes. ECOG PS > 0 has a negative prognostic impact on PFS and OS, in line with previous studies with CG and CGD,^25^ and baseline elevated CA 19-9 levels are associated with shorter PFS. Of note, in a prospective analysis on a cohort of 267 patients, patients with tumor marker response more frequently had a radiological response and better outcomes,^26^ making CA 19-9 response potentially useful in clinical practice.

Our research has several limitations. It is a retrospective investigation with possible confounding factors in the included cohorts. Moreover, due to the multicentric nature of the study, PFS, ORR, and DCR data have to be contextualized and a slight difference in tumor assessment modalities and time points among different institutions has to be considered. A longer observation period and further prospective studies are needed to confirm our results.

In conclusion, to the best of our knowledge, we reported the results of the first real-world study of first-line CGD in patients with BTC with early relapse. Our analysis suggests that this group of patients, similar to the group with a later recurrence, can benefit from chemoimmunotherapy in terms of OS, PFS, and ORR and without a significant difference in toxicity. Therefore, based on our findings, patients who experience early relapse may be considered for inclusion in future clinical trials investigating novel first-line systemic approaches in BTC and for adding Durvalumab to cisplatine-gemcitabine in routine clinical practice.

## Supplementary material

Supplementary material is available at *The Oncologist* online.

oyae256_suppl_Supplementary_Table_1

oyae256_suppl_Supplementary_Table_2

oyae256_suppl_Supplementary_Table_3

## Data Availability

Data available on request from the authors.
