# Supplementary material for: Efficacy of cisplatin-gemcitabine-durvalumab in patients with advanced biliary tract cancer experiencing early vs late disease relapse after surgery: a large real-life worldwide population
Source: Oncologist. 2024 Oct 19;30(3):oyae256. doi: 10.1093/oncolo/oyae256 (PMC11954499; doi:10.1093/oncolo/oyae256)
Supplement: oyae256_suppl_Supplementary_Table_1 [file oyae256_suppl_supplementary_table_1.docx]

|  | **Relapse and started systemic therapy** ≤ **6 months**  N=77 | **Relapse and started systemic therapy >6 months**  N=80 | P |
| --- | --- | --- | --- |
| Best Overall Response  CR  PR  SD  PD | 8 (10.3)  18 (23.3)  34(44.1)  17(22.0) | 3(3.7)  25(31.2)  38(47.5)  14(17.5) |  |
| ORR | 26(29.8) | 28 (30.7) | 1.0 |
| DCR | 60 (68.9) | 66(72.5) | 0.62 |

*Supplementary Table 1: ORR and DCR according to disease relapse* ≤*6 months and >6 months.*
