# Supplementary material for: Efficacy of cisplatin-gemcitabine-durvalumab in patients with advanced biliary tract cancer experiencing early vs late disease relapse after surgery: a large real-life worldwide population
Source: Oncologist. 2024 Oct 19;30(3):oyae256. doi: 10.1093/oncolo/oyae256 (PMC11954499; doi:10.1093/oncolo/oyae256)
Supplement: oyae256_suppl_Supplementary_Table_2 [file oyae256_suppl_supplementary_table_2.docx]

| **Adverse Event** | **Relapse and started systemic therapy< 6 months**  **N=87** | **Relapse and started systemic therapy> 6 months**  **N=91** | **p** |
| --- | --- | --- | --- |
| **Colangitis**  0-2  >2  Not Reported | 80(91.9)  1(1.1)  6(6.8) | 86(94.5)  1(1.0)  4(4.3) | 1.0 |
| **Colitis**  0-2  >2  Not Reported | 79(90.8)  1(1.1)  7(8.0) | 86(94.5)  0(0)  5(5.4) | 0.48 |
| **Hypothyroidism**  0-2  >2  Not Reported | 80(91.9)  0(0)  7(8.0) | 86(94.5)  0(0)  5(5.4) | - |
| **Hyperthyroidism**  0-2  >2  Not Reported | 80(91.9)  0(0)  7(8.0) | 86(94.5)  0(0)  5(5.4) | - |
| **Rash**  0-2  >2  Not Reported | 79(90.8)  0(0)  8(9.1) | 85(93.4)  1(1.0)  5(5.4) | 1.0 |
| **Itching**  0-2  >2  Not Reported | 79(90.8)  0(0)  8(9.1) | 86(94.5)  0(0)  5(5.4) | - |
| **Other immune-mediated toxicities**  0-2  >2  Not Reported | 78(89.6)  1(1.1)  8(9.1) | 86(94.5)  0(0)  5(5.4) | 0.47 |
| **Neuropathy**  0-2  >2  Not Reported | 80(91.9)  1(1.1)  6(6.8) | 87(95.6)  2(2.1)  2(2.1) | 1.0 |
| **Diarrhea**  0-2  >2  Not Reported | 80(91.9)  0(0)  7(8.0) | 84(92.3)  2(2.2)  5(5.4) | 0.49 |
| **Thrombocitosis**  0-2  >2  Not Reported | 83(95.5)  0(0)  4(4.5) | 88(96.7)  1(1.0)  2(2.1) | 1.0 |
| **Vomiting**  0-2  >2  Not Reported | 78(89.6)  2(2.2)  7(8.0) | 86(94.5)  4(4.3)  1(1.0) | 0.68 |
| **Constipation**  0-2  >2  Not Reported | 78(81.6)  1(1.1)  8(9.1) | 87(95.6)  0(0)  4(4.3) | 0.47 |
| **Fever**  0-2  >2  Not Reported | 81(93.1)  0(0)  6(6.8) | 87(95.6)  0(0)  4(4.3) | - |
| **Leukopenia**  0-2  >2  Not Reported | 76(87.3)  6(6.8)  5(5.7) | 84(92.3)  6(6.5)  1(1.0) | 1.0 |
| **Nausea**  0-2  >2  Not Reported | 79(90.8)  1(1.1)  7(8.0) | 83(91.2)  3(3.2)  5(5.4) | 0.62 |
| **ALT increased**  0-2  >2  Not Reported | 78(89.6)  0(0)  8(9.1) | 86(94.5)  0(0)  5(5.4) | - |
| **Neutropenia**  0-2  >2  Not Reported | 60(68.9)  23(26.4)  4(4.5) | 65(71.4)  25(27.4)  1(1.0) | 1.0 |
| **Anemia**  0-2  >2  Not Reported | 74(85.0)  5(5.7)  8(9.1) | 84(92.3)  4(4.3)  3(3.2) | 0.73 |
| **Fatigue**  0-2  >2  Not Reported | 80(91.9)  1(1.1)  6(6.8) | 85(93.4)  4(4.3)  2(2.1) | 0.37 |
| **Thrombocytopenia**  0-2  >2  Not Reported | 74(85.0)  8(9.1)  5(5.7) | 84(92.3)  5(5.4)  2(2.1) | 0.39 |
| **Other toxicity**  0-2  >2  Not Reported | 79(90.8)  1(1.1)  7(8.0) | 87(95.6)  1(1.0)  3(3.2) | 1.0 |
