# Supplementary material for: Efficacy of cisplatin-gemcitabine-durvalumab in patients with advanced biliary tract cancer experiencing early vs late disease relapse after surgery: a large real-life worldwide population
Source: Oncologist. 2024 Oct 19;30(3):oyae256. doi: 10.1093/oncolo/oyae256 (PMC11954499; doi:10.1093/oncolo/oyae256)
Supplement: oyae256_suppl_Supplementary_Table_3 [file oyae256_suppl_supplementary_table_3.docx]

|  | **Relapse and started Cisplatin-gemcitabine** ≤ **6 months**  **N=59** | **Relapse and started cisplatin-gemcitabine-durvalumab**  ≤**6 months**  **N=77** | **p** |
| --- | --- | --- | --- |
| Best Overall Response  CR  PR  SD  PD | 4 (6.7)  14 (23.7)  19(32.2)  22(37.2) | 8 (10.3)  18 (23.3)  34(44.1)  17(22.0) |  |
| ORR | 18(30.5) | 26 (29.8) | 1.0 |
| DCR | 37 (62.7) | 60(68.9) | 0.47 |

*Supplementary Table* ***3a*** *ORR and DCR in patients with disease relapse and treatment initiation (cisplatin-gemcitabine and cisplatin-gemcitabine-durvalumab)* ≤6*months after surgery/completion of adjuvant therapy.*
